# Restored forested wetland surprisingly resistant to experimental salinization

**DOI:** 10.1371/journal.pone.0296128

**Published:** 2023-12-21

**Authors:** Emily A. Ury, Marcelo Ardón, Justin P. Wright, Emily S. Bernhardt

**Affiliations:** 1 Department of Biology, Duke University, Durham, North Carolina, United States of America; 2 Department of Forestry and Environmental Resources, North Carolina State University, Raleigh, North Carolina, United States of America; ICAR-National Rice Research Institute, INDIA

## Abstract

Salinization of coastal freshwater wetlands is an increasingly common and widespread phenomenon resulting from climate change. The ecosystem consequences of added salinity are poorly constrained and highly variable across prior observational and experimental studies. We added 1.8 metric tons of marine salts to replicated 200 m^2^ plots within a restored forested wetland in Eastern North Carolina over the course of four years. Based on prior small-scale experiments at this site, we predicted that salinization would lead to slower tree growth and suppressed soil carbon cycling. Results from this large-scale field experiment were subtle and inconsistent over space and time. By the fourth year of the experiment, we observed the predicted suppression of soil respiration and a reduction of water extractable carbon from soils receiving salt treatments. However, we found no cumulative effects of four years of salinization on total soil carbon stocks, tree growth, or root biomass. We observed substantial variation in soil solution chemistry (notably, pH and base saturation) across replicated treatment blocks; the effective salt levels, ionic composition, and pH varied following treatment depending upon pre-existing differences in edaphic factors. Our multi-year monitoring also revealed an underlying trend of wetland acidification across the entire site, a suspected effect of ecosystem recovery following wetland restoration on former agricultural land. The overwhelming resistance to our salt treatments could be attributed to the vigor of a relatively young, healthy wetland ecosystem. The heterogeneous responses to salt that we observed over space and time merits further investigation into the environmental factors that control carbon cycling in wetlands. This work highlights the importance of multi-year, large-scale field experiments for investigating ecosystem responses to global environmental change.

## Introduction

In coastal regions, climate change is rapidly altering the boundaries between freshwater and saltwater ecosystems. While much attention has been paid to the gradual loss of coastal margins due to sea level rise, the salinization of coastal freshwaters is a more pervasive, yet cryptic, consequence [1]. Both droughts and storm surges exacerbate the introduction of marine salts to inland wetland ecosystems, leading to dramatic changes in soil chemistry [1, 2]. Freshwater forested wetlands have historically dominated coastal landscapes of low topographic relief, like the extensive North Atlantic Coastal Plain (NACP) and these ecosystems are proving particularly vulnerable to rising water tables and increasing salinity. Several recent studies have documented the loss of forested wetlands along both the Atlantic [3–5] and Gulf [6] coasts. White and others [7] documented a loss of more than 8% of coastal forested wetlands across the entire NACP since 1996. These losses are rapid even within protected areas and suggest that despite efforts to conserve these highly threatened ecosystems, climate change driven deforestation is now a primary threat to their protection.

Coastal forested wetlands are uniquely important ecosystems. The plants and animals supported in coastal forested wetlands are distinct from coastal salt marsh, but they still provide many of the same valuable ecosystem services, including coastal protection, habitat provisioning, water filtration and long-term carbon storage [8]. The loss of freshwater wetland trees leads to substantial restructuring of the plant community [9, 10] and dramatic reductions in aboveground biomass [11]. Smart and others [4] documented a 28% loss of biomass in forests of Eastern Carolina, associated with sea level rise.

While the effects of salinization plant communities are well documented, the consequences for belowground carbon storage are far less clear. Ecosystem responses to elevated salinity are complex because sea water’s many constituents (chloride, sulfate, sodium, other base cations, and trace minerals) have numerous direct and indirect effects on organisms and wetland chemistry [2, 12–14]. Base cation build-up following repeated exposure to salt water may alter the alkalinity status of soils, impacting soil microbial functioning [15]. Sulfidation, another result of seawater exposure in wetlands, is also harmful to many taxa, but may be of less concern in some iron rich soils, such as those in Eastern North Carolina [16]. Additionally, there are plant-soil feedbacks that indirectly affect ecosystem responses to salinization and have not been well studied in freshwater wetlands [17]. Both vegetation and edaphic factors may mediate the effect of salinity on soil microbial functions, effects that are difficult to study in a laboratory setting or small field studies.

Previous salt addition experiments show a surprising amount of disagreement with respect to carbon cycling responses to salinity, which may be in part due to a lack of understanding of key environmental covariates. Several salt addition experiments have shown salt suppression of carbon mineralization [18–20], while others show salt stimulation of soil respiration [21–24]. The interactions between salinization and carbon availability within soil pore water may indirectly affect carbon mineralization rates beyond the direct effects of osmotic stress on soil microbes [25, 26]. Vegetation may play an important role in the mediation of salinization effects on soil carbon processing but is often left out of experimental design for sake of reducing study complexity. Studies that do include vegetation are typically conducted in salt marshes or in mesocosms with herbaceous plants, occasionally saplings, but seldom if ever, with full grown trees [17]. To date, no large-scale, long-term field salinization experiments have been conducted in forested freshwater wetlands, despite these ecosystems being highly vulnerable to coastal change.

Wetland restoration and creation is expanding in coastal regions as farm profitability in low-lying areas declines [27]. Restoring coastal plain wetlands helps improve water quality, promote biodiversity, and protect from coastal storms, among other ecosystem services [28, 29]. Wetland restoration can also provide supplemental income to farmers through wetland mitigation banking and water quality trading [30] as well as emerging enterprises for voluntary carbon offsets [31]. There is, however, considerable uncertainty surrounding not only the efficacy of wetland restoration for delivering the desired ecosystem services, but also the fate of carbon stored in freshwater wetlands vulnerable to sea level rise [32, 33]. Understanding the effects of salinization on carbon in restored freshwater wetlands is urgently needed.

We established the first large-scale salt addition experiment in a restored freshwater forested wetland to address the question of how salinization affects ecosystem carbon stocks and cycling. The aim of this study is to assess ecosystem responses to marine salt addition in a field setting large enough to affect the rooting zones of numerous mature trees. Our objectives are to track above- and belowground biomass response to elevated salinity as well as soil carbon responses, specifically soil microbial respiration, soil carbon lability, and total soil carbon content. We aim to leverage the large and multi-year nature of the experiment to quantify both spatial and temporal variability in the responses to salt additions. We hypothesize that salinity would adversely affect vegetation associated carbon pools leading to reduced tree growth and root biomass. We hypothesize that salt would suppress microbially mediated soil carbon cycling resulting in reduced rates of soil carbon mineralization. We anticipate that the salt treatment would also reduce soil carbon lability due to increased alkalinity in the soil and lead to an overall increase in bulk soil carbon content.

## Materials and methods

### Site description and experimental design

Salt addition plots were established in 2015 in the Timberlake Observatory for Wetland Restoration (TOWeR, see Ardón et al. [34] for additional details about the property) located on the coastal plain of North Carolina (35°54′22″ N, 76°09′25″ E, elevation < 3 meters above mean sea level). The property is a restored wetland on formerly agricultural land that underwent rewetting and revegetation in 2004 with the planting of 750,000 native wetland tree saplings. The site is 8 km from the shore of the Albemarle Sound and surface water typically has very low salinity (<0.5 ppt or 1.0 dS·m^-1^). However, during major drought events, salinity in the channel has been observed to reach over 5 ppt (9.0 dS·m^-1^) due to saltwater incursion [35]. There are two main soil series on the property, Hyde loam and Ponzer muck. The dominant tree species are *Taxodium distichum*, *Pinus taeda*, *Salix nigra*, *Liquidambar styraciflua*, and several *Quercus sp*. all of which have low to moderate salt tolerance [36]. Permission to access the site for research purposes was provided by the landowner, the Great Dismal Swamp RestorationBank, Limited Liability Company.

**Fig 1:**
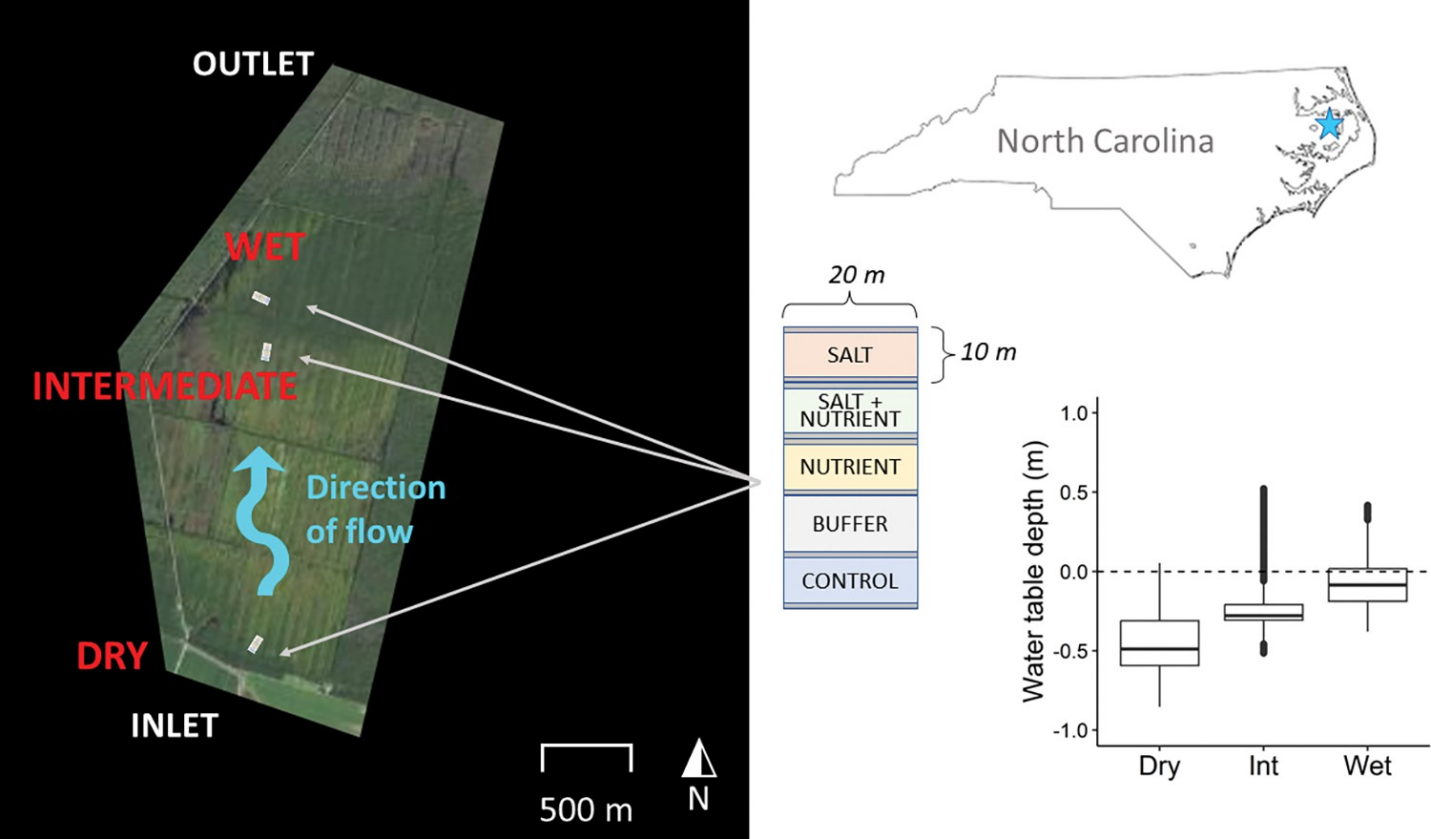
Site map and experimental design. TOWeR site map with experimental site locations (left). Location of TOWeR in North Carolina (top right) and schematic layout of salt and nutrient addition plots (center). Note that data from the nutrient addition plots are not reported in this manuscript. Each experimental plot is 10 x 20 meters including a 1-meter buffer strip which was included in the treatment but excluded from sampling to avoid edge effects. A full 10-meter buffer plot separates treatment plots from control plots. The inset boxplot shows the distribution of water level measurements taken between 2017 and 2020 within the wells (averaged across each site). The ariel photograph base map is from the National Agriculture Imagery Program (NAIP), Digital Object Identifier (DOI) number: /10.5066/F7QN651G.

A set of salt and control treatment plots were established at three sites, spread out across the property (Fig 1). A small elevation gradient (<1 m) exists across the property such that the sites at the northern end are more frequently inundated than the site at the southern end. Because of this variation in hydrology, we have monikered the sites as ‘dry’, ‘intermediate’ and ‘wet’, which refers to their average conditions relative to one another, although all locations are considered wetlands. Consistent with the hydrologic gradient, there is some variation in the species of trees planted across the site, wherein the wet site is more dominated by *Taxodium distichum* and *Pinus taeda*, the intermediate site is mainly *Quercus* species and the driest site a mix of *Liquidambar styraciflua* and *Quercus* species. Each site is dominated by soil from a different series. The wettest site is Hyde loam (fine-silty, mixed, active, thermic *Typic Umbraquults*), the intermediate site is Ponzer muck (loamy, mixed, dysic, thermic *Terric Haplosaprists*), and the dry site is Weeksville silt loam (coarse-silty, mixed, semiactive, acid, thermic *Typic Humaquepts*) [37, 38]. A set of PVC monitoring wells was established within each treatment plot, equipped with Onset U20L HOBO water level data loggers and conductivity sensors, each logging on 15-minute time steps.

Each salt treatment plot is 10 by 20 meters and was established at least 10 meters away from a control plot of the same size. Salt additions began in the fall of 2016 using Instant Ocean® marine aquarium salt mix, applied dry. Instant Ocean® is a complete sea water replacement containing chloride, sodium, sulfate, magnesium, calcium, potassium, and several other trace minerals in ratios similar to natural sea water. Care was taken to ensure an even application of salt across the plot and salt was added at a rate of 68 grams per m^2^ between 3–9 times each year (see S1 Fig and S1 Table for a schedule of field activities). Sampling was conducted approximately one month following the most recent salt application except for summer 2020 when travel restrictions due to Covid-19 forced us to sample only 4 days following the final salt application.

### Vegetation monitoring

Prior to the first salt addition, trees larger than 2 cm in diameter within the treatment plots were identified, tagged, and measured (diameter at breast height, DBH) in fall 2015. A maximum of 10 individuals of a single species within each plot were tagged and measured. Tagged trees were re-measured (DBH) in January 2021. Roots collected in soil cores (described below) were cleaned, dried and weighed during each sampling period.

### Soil collection and analysis

Soil cores were collected from the salt and control plots four times: May and July of 2018, June 2019, and August 2020. At each sampling, 5 soil cores (5 cm diameter) were collected from a randomly selected transect across the plot. Soil cores, in plastic sleeves, were capped and kept on ice during transport back to the lab where they were stored at 4°C, processed, and analyzed within 72 hours.

Soils cores were extruded from their sleeves and sectioned into two depth increments: 0–5 cm and 5–10 cm. The weight of each core section was recorded for estimation of wet bulk density. Each increment was passed through a 2 mm sieve to remove roots, rocks, and large organic debris. Sieved soil was homogenized and stored in sterile plastic bags. Subsamples of each core section were weighed on an analytical balance for the following analyses and all results have been corrected by soil mass. Approximately 10 g of soil was weighed in an aluminum tin for measuring soil moisture and organic content by mass difference after 48 hours in a drying oven at 60°C followed by 4 hours in a muffle furnace at 500°C. A 5 g subsample was weighed into a conical tube for measuring pH in a soil-water slurry with a 1:2 ratio using a calibrated hand-held probe device (Hach H260G pH Meter, Loveland, CO).

Five grams of soil each were weighed into amber glass I-Chem^TM^ vials for carbon mineralization and substrate induced respiration (SIR) assays (carbon mineralization assays were conducted from July 2018 onward). Accumulated CO_2_ was measured on a LI-6250 flow-through gas analyzer (Li-core Inc., Lincoln, Nebraska, USA) following protocols modified from Fierer et al. [39] and described in detail in Marinos and Bernhardt [40]. In brief, vials were sealed with a PFTE coated silicon septa and gas was allowed to accumulate for 24 hours before sampling. The headspace was sampled with a 1-mL glass syringe and injected into the port of the flow-through analyzer. After sampling, vials were vented to prevent anoxia. Vials were recapped 24 hours prior to subsequent sampling on days 3 and 7 of the incubation period. SIR assays were conducted in the same way, with the addition of 10 mL of autolyzed yeast solution. Vials remained sealed for the duration of the SIR assay and were kept aerated via mixing on a shaker table. One mL of CO_2_ free air was injected into each vial prior to sampling to maintain positive pressure within the vial and samples were drawn and analyzed at 10 minutes, 2 hours, and 4 hours following substrate addition and sealing.

Four-gram aliquots of soil were weighed into conical tubes for water extraction using a 1:10 soil to water ratio. Soil slurries were shaken on an end-over-end rotating table at 60 rpm for 4 hours. Slurries were allowed to settle overnight at 4°C and then centrifuged at 3500 rpm for 15 minutes. The supernatant was carefully poured off and passed through a 0.7 μm glass fiber filter. Filtrates were analyzed for ion content and dissolved organic carbon at the Duke River Center on a Dionex ICS-2000 Ion Chromatograph (Sunnyvale, CA) and a TOC-V combustion analyzer (Shimadzu Corporation, Kyoto, Japan). Filtrate was also analyzed for phenolic compounds using a colorimetric method described in Ohno and First [41]. In brief, 0.1 mL of Folin-Ciocalteu’s Reagent and 0.30 mL of 0.5 M NaHCO_3_ were added to 1 mL of soil water extract and gently mixed before allowing color to develop for 4 hours. UV-vis absorbance spectra were measured on a BioTek Epoch^TM^ 2 Microplate Spectrophotometer (Winooski, VT, USA) at 750 nm and calibrated against a vanillic acid standard (0 to 10 mg·L^-1^). The concentration of phenolic compounds is also reported as mg·mg^-1^ DOC after normalizing for the concentration of DOC in each extract following Weishaar *et al*. [42].

A separate soil sampling campaign was conducted on July 2, 2020, for further soil testing and analysis conducted by The North Carolina Division of Agriculture and Consumer Services (NCDA&CS). Air-dried samples were tested for pH (1:1 soil to water ratio by volume), humic matter (NaOH digestion with colorimetric determination), base saturation, cation exchange capacity, and exchangeable acidity [43, 44].

### Statistical analyses

Statistical analyses were conducted in the R statistical computing environment [45] with the ‘tidyverse’ [46], ‘ggplot2’ [47], and ‘cowplot’ [48] packages. Ion concentration data (Cl^-^, Na^+^, Mg^2+^, Ca^2+^, K^+^, SO_4_^2-^) were log transformed to improve normality and simple linear regression (R function ‘lm()’) was used to assess correlations between soil characteristics. Soil ion content and bulk density were used to assess the retention efficiency of the salt addition treatment within each treatment plot. Percent retention is calculated as the proportion of the total mass of salt added over the duration of the experiment retained in the top five centimeters of the soil. Soil bulk density and the ion concentration measured in 2020 (mg·kg^-1^ dry soil) were used to calculate the mass of salt recovered, adjusting for the soil ion content of the control plots (in lieu of pre-experiment soil salt content).

Pairwise comparisons were made between the salt and control plots using one-way Analysis of Variance (ANOVA, R function ‘aov()’) for soil characteristics and response variables within a given sampling date, site, and depth. Limited sample size and the irregularity of the salt addition schedule made it infeasible to perform an interannual comparison of treatment responses.

Principal components analysis (PCA, R function ‘prcomp()’) was used to compare the ion assemblage across experimental sites. Principal Components Analysis (PCA) is used to visualize how the composition of the extractable soil ion content has shifted in the salt treatment plots as compared to the control plots. PCA results were interpreted in the context of the soil properties measured by NCDA&CS, where differences between treatment and control plots were calculated for each variable (*Var*) on a percent basis as:

$$Percent\;difference=\frac{\left({Var}_{Treatment}-{Var}_{Control}\right)}{{Var}_{Treatment}}\;x\;100$$ (1)

## Results

Median water table depth within each site over the duration of the experiment was 0.085 m in the wet site, 0.28 m in the intermediate site, and 0.49 m in the dry site. Water level fluctuated significantly over time; the distribution of well water level measurements is shown in Fig 1.

### Variable efficacy of salt addition treatments

**Fig 2:**
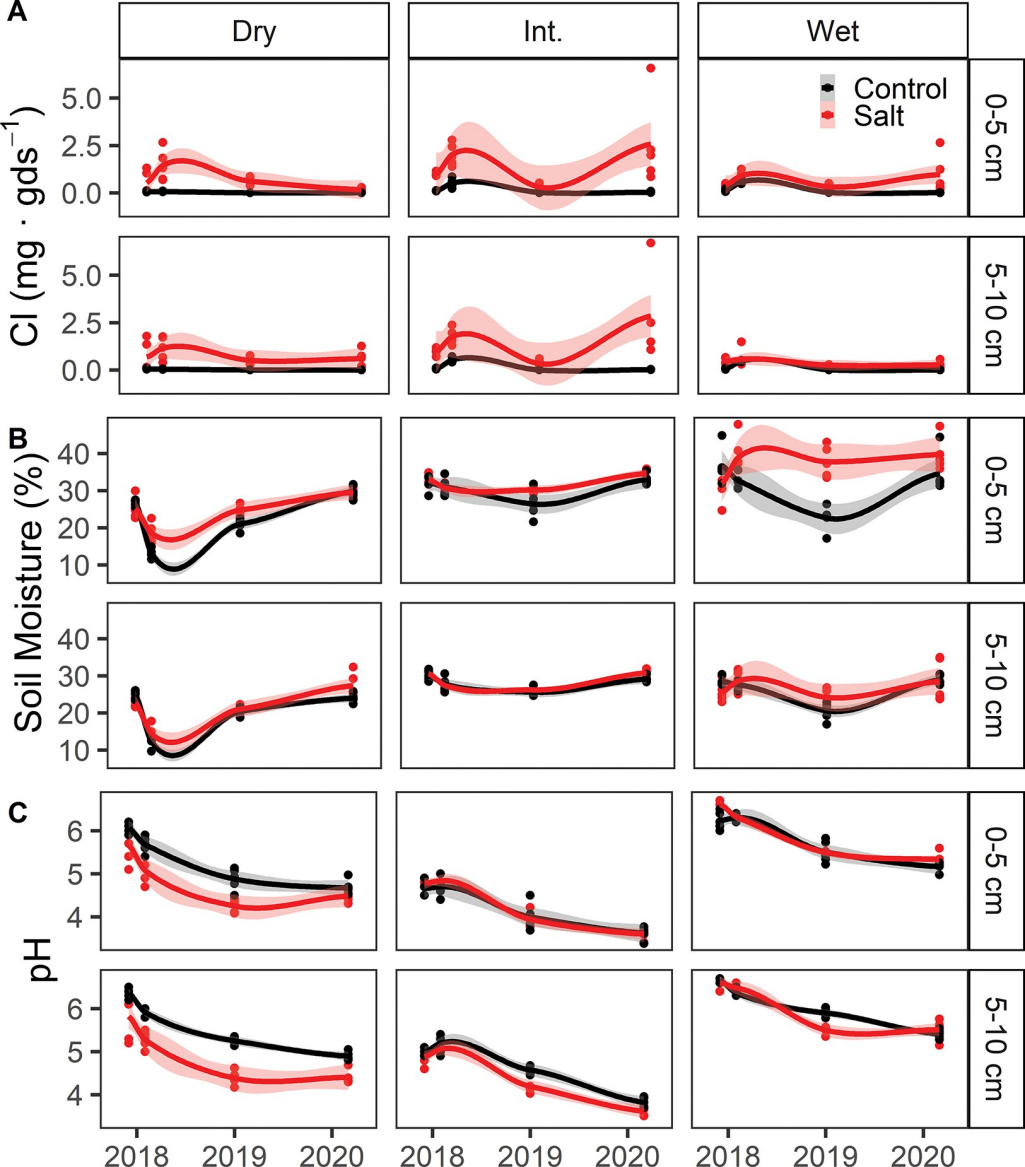
Efficacy of salt addition treatments across sites. (A) Water extractable soil chloride content, (B) soil moisture, and (C) soil pH, for each sampling date, site and soil depth increment. Trend lines depict Loess-smoothed local polynomial regression and standard error shading.

All three experimental sites (dry, intermediate, and wet) received salt additions according to the same schedule (S1 Table), but the efficacy of the salt treatments was highly variable across sites and over time (see S2 Table for pairwise comparisons between salt and control plots for all measurements). Fig 2 illustrates the soil chloride enrichment achieved by the salt addition treatments at the time of soil sampling. The intermediately inundated plot achieved the highest soil chloride levels, but also exhibited large variability between sampling dates. Soil salt content did not build up gradually over time as we had expected, nor was it contingent on soil moisture. Soil moisture at the time of sampling showed a very weak positive correlation (R^2^ = 0.04, p = 0.001) with soil chloride content within the experimental plots (Fig 2). Continuous conductivity data from sensors mounted in sampling wells demonstrate the variability of salinity measured within the pore water of the experimental plots (S1 Fig). We suspect that cumulative precipitation (rather than soil moisture at the time of sampling) was driving patterns in soil salt content over time.

In general, salt treatments elevated soil salinity, but the degree of salinization achieved also varied across each of the constituent ions within the marine salt mixture. By the end of the experiment, an average of 12% of the added salts remained in the top 5 cm of the soil (**Table 1**). Potassium and sulfate were the most readily retained, but retention is highly variable across the three sites. Calcium was conserved in the wet site (20%) but in the dry site there was less water extractable calcium in the salt treatment plot than in the control plot, resulting in a negative percent retention (-9%).

**Table 1:** Soil ion content.

	2020		Recovery of total ion added (% by mass) in the top 5 cm		
*(mg*·*kg*^*-1*^*)*	Control	Salt	Dry	Int.	Wet
Cl^-^	18.9 (5.4)	1240 (439)	1.5	23.4	8.9
SO_4_^2-^	15.4 (1.9)	218 (69)	5.3	32.2	9.4
Na^+^	27.0 (3.9)	785 (252)	2.4	24.5	9.5
K^+^	6.0 (1.4)	40.1 (11)	5.6	29.9	12.8
Ca^2+^	22.0 (3.2)	40.0 (13)	-9.4	13.7	19.6
Mg^2+^	1.8 (0.4)	78.1 (31)	0.67	23.2	7.0

Soil ion content in 2020 from salt and control plots, 0–5 cm depth only, averaged across sites (standard error in parentheses) and percent recovery of each ion at each site.

**Fig 3:**
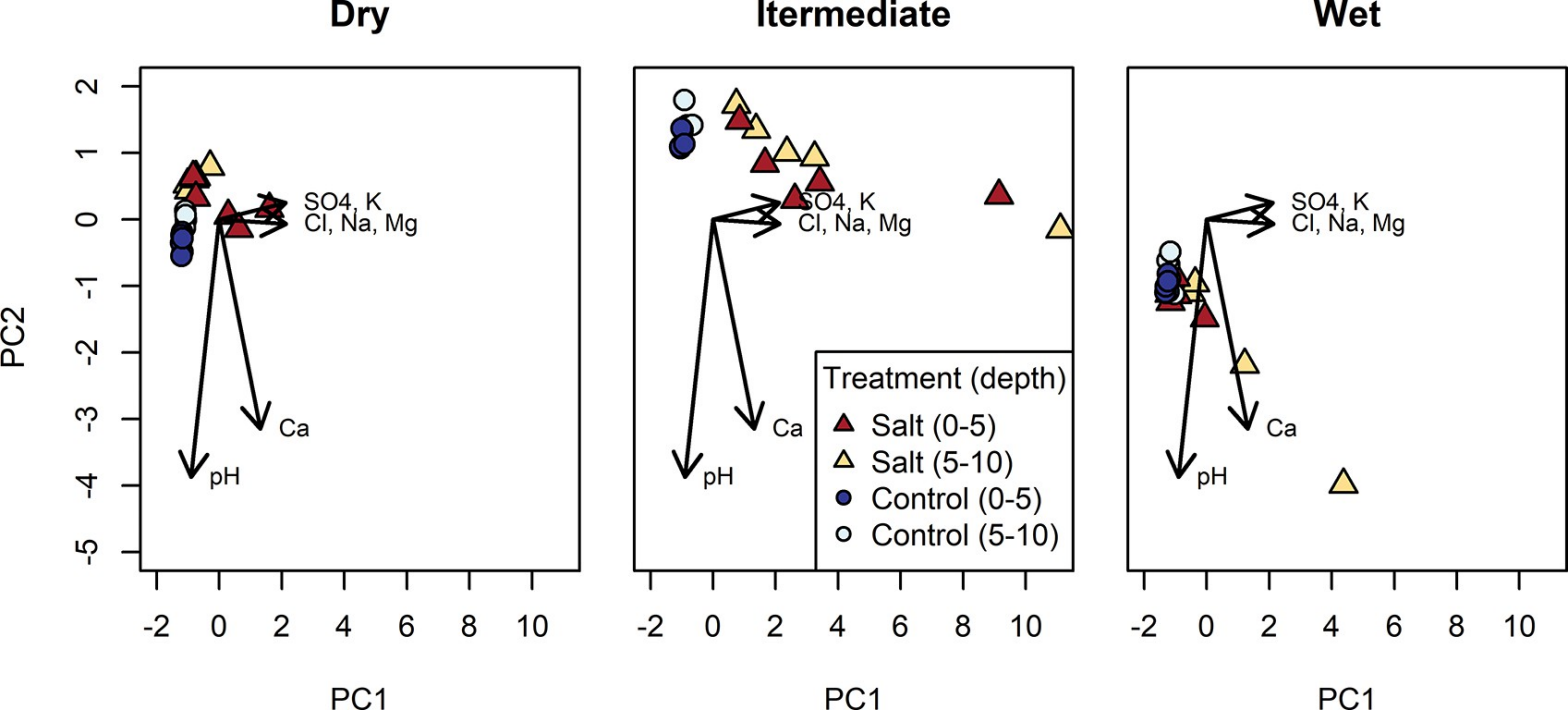
PCA of soil ion content. Soil samples from the salt treatment (triangles) and control (circles) plots, across all three sites from samples collected in August 2020. Soil sample depth is indicated by color, darker shades corresponding to the shallow sampling depth (0–5 cm) and lighter shades, the deeper depth (5–10 cm). Variables contributing to the PCA analysis include the soil content (water extractable) of major ions (Cl^-^, SO_4_^2-^, Na^+^, K^+^, Mg^2+^, and Ca^2+^) plus soil pH. Arrows indicate the contribution of each soil ion with respect to the principal components, where arrow length represents the strength of the loading.

Principal Component Analysis (PCA) demonstrates a shift in extractable soil ion content in the salt treatment plots relative to the control plots (Fig 3). Differences in soil calcium and pH drive separation between the dry, intermediate and wet sites in PCA space, primarily in the y-direction (PC2). Differences between the control and salt treatment soils are more apparent in the x-direction (PC1), driven by the presence of chloride, sodium, sulfate, potassium, and magnesium. The dry site shows minimal separation between the control and salt treatment plots, consistent with lower ion retention (Table 1). The intermediate site shows the greatest separation between salt and control plots. The wet site shows separation between salt and control plots along both PCA1 and PCA2, the latter being driven by strong calcium retention observed in the salt treatment plot at this site (Table 1).

We anticipated that the salt addition treatment would elevate soil pH due to the addition of base cations, but this was not the case in the majority of sites and sampling dates. At the driest site, the salt treatment lowered the pH relative to the control plots, but no consistent pattern was observed at the other two sites (Fig 2). We observed a surprising decline in pH overtime across all sites and treatments (Fig 2), a trend which may be interacting with the overall treatment effect on pH as well as on other soil properties and treatment responses.

Difference in pH is not the only source of variability between the three sites. Detailed soil analysis from NCDA&CS shows that humic matter content and cation exchange capacity are relatively consistent across sites, but base saturation and exchangeable acidity vary substantially (Table 2). The wet site exhibits the highest base saturation of all three sites and the lowest exchangeable acidity. These edaphic factors are not directly affected by the salt treatment, but they can alter the way the salt ions interact within the soil matrix and in turn the effect the salt treatment has on soil living organisms and biogeochemical processes.

**Table 2:** Soil characteristics.

Measurement	Site	Depth (cm)	Control	Salt	% Diff^1^
pH	Dry	0–5	5.0 (0.075)	4.9 (0.051)	-2.0
		5–10	5.3 (0.093)	5.0 (0.02)	-5.7*
	Int.	0–5	4.0 (0.024)	4.3 (0.051)	7.5*
		5–10	4.2 (0.024)	4.3 (0.032)	2.4
	Wet	0–5	5.6 (0.058)	5.7 (0.024)	1.8
		5–10	5.8 (0.024)	5.8 (0.037)	0
Humic matter (%)	Dry	0–5	1.9 (0.08)	1.9 (0.04)	0
		5–10	2.1 (0.07)	2.2 (0.06)	4.8
	Int.	0–5	2.9 (0.11)	3.1 (0.14)	6.9
		5–10	3.3 (0.09)	3.9 (0.09)	18.2*
	Wet	0–5	2.2 (0.08)	1.7 (0.2)	-22.7
		5–10	2.5 (0.05)	1.7 (0.17)	-32.0*
Base saturation (%)	Dry	0–5	68.0 (2.5)	52.6 (2.0)	-22.6*
		5–10	75.0 (2.4)	54.0 (1.1)	-28.0*
	Int.	0–5	17 (0.9)	28.8 (3.6)	69.4*
		5–10	14.8 (0.7)	33.6 (2.3)	127.0*
	Wet	0–5	78.2 (1.8)	81.4 (0.9)	4.1
		5–10	81.6 (1.6)	80.4 (1.2)	-1.5
Cation exchange capacity (meq·100cm^-3^)	Dry	0–5	8.2 (0.31)	6.5 (0.26)	-20.7*
		5–10	8.5 (0.44)	5.5 (0.14)	-35.3*
	Int.	0–5	6.3 (0.32)	7.4 (0.18)	17.5*
		5–10	4.7 (0.06)	6.0 (0.14)	27.7*
	Wet	0–5	7.9 (0.22)	9.4 (0.7)	19.0
		5–10	8.2 (0.14)	7.5 (0.64)	-8.5
Exchangeable acidity (meq·100cm^-3^)	Dry	0–5	2.6 (0.11)	3.1 (0.19)	19.2*
		5–10	2.1 (0.11)	2.5 (0.05)	19.0*
	Int.	0–5	5.2 (0.28)	5.3 (0.20)	1.9
		5–10	4.0 (0.07)	4.6 (0.12)	15.0*
	Wet	0–5	1.7 (0.10)	1.7 (0.06)	0
		5–10	1.5 (0.11)	1.5 (0.13	0

Mean (standard deviation) and percent difference between treatment plots for soil characteristics from soils collected in July 2020. Soil analyses conducted by NCDA&CS.

^1^Significant difference between groups marked with * (p < 0.05, Mann Whitney U-Test)

### Salt treatment effects on wetland carbon

We examined the effect of salt treatment on soil carbon pools (bulk carbon and aromaticity of organic carbon) and fluxes (carbon mineralization and solubility of organic carbon) as well as vegetation associated carbon pools (tree and root biomass). We found no consistent effect of the salt treatment across all three sites and over time in any measured response variables.

**Fig 4:**
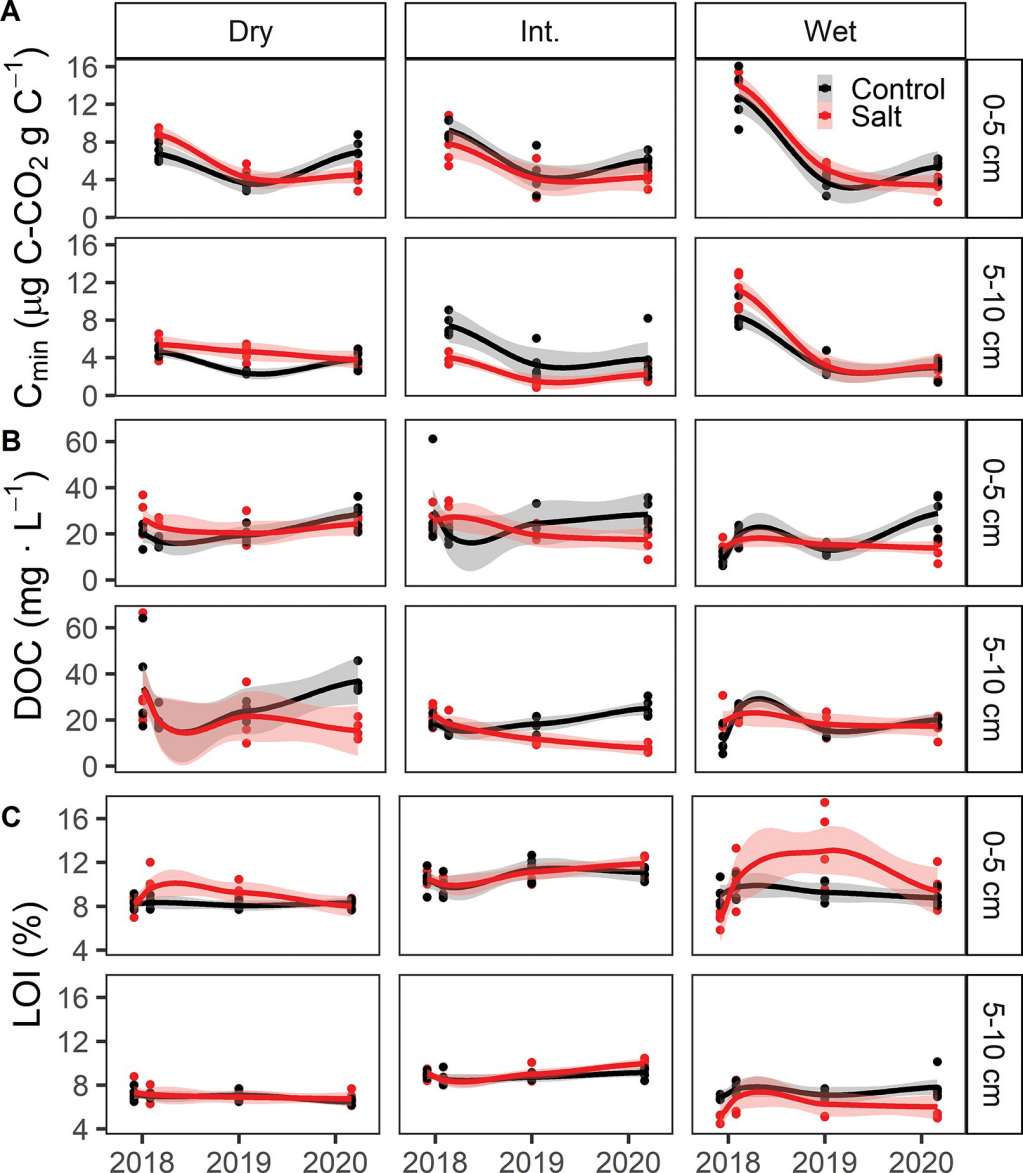
Soil carbon responses to salt treatments. (A) Soil carbon mineralization potential, (B) water extractable dissolved organic carbon (DOC), and (C) bulk soil carbon measured as loss on ignition (LOI) for each sampling date, site, and depth. Trend lines depict Loess-smoothed local polynomial regression and standard error shading.

Carbon mineralization was suppressed by the salt treatment at the time of the final sampling (Fig 4), but the degree of suppression was highly variable across sites. Carbon mineralization rates ranged from 1.5 to 14.0 μg CO_2_-C·hr^-1^ per gram carbon and from 0.14 to 1.5 μg CO_2_-C·hr^-1^ on a per gram dry soil on basis (S2 Fig). Respiration rates in soils from the deeper core section were consistently lower than soils from the shallower depth.

Salt treatments reduced water extractable dissolved organic carbon compared to control plots at all sites during the final sampling period, however the effect was not statistically significant across both depths (S2 Table). The overall range of DOC concentrations was 7.76 mg·L^-1^ (August 2020, intermediate site, salt treatment plot, 5–10 cm) to 36.8 mg·L^-1^ (August 2020, dry site, control plot, 5–10 cm). We examined the phenolic content of extractable DOC as a proxy for aromaticity or the lability of this leachable carbon pool. The range of phenolic compound concentrations observed was 0.59 mg·L^-1^ (August 2020, intermediate site, salt treatment plot, 5–10) to 7.68 mg·L^-1^ (May 2018, dry, control plot, 0–5). There were less phenolics in the DOC from the salt treatment plots during the final sampling period, but no other consistent results across sites (S3 Fig). We also observed a general decline in phenolics over time across all sites and treatment plots, a trend consistent with declining soil pH, which may be driving declining solubility of large, organic molecules.

These subtle and variable salt treatment effects did not accumulate to any measurable impacts on total bulk soil carbon content, even after four years (Fig 4). We also use a substrate induced respiration (SIR) assay as a proxy for carbon associated with soil living microorganism, or microbial biomass. SIR ranged from 0.17 to 9.8 CO_2_-C μg·hr^-1^ per gram dry soil and 1.5 to 105 CO_2_-C μg·hr^-1^ on a per gram carbon basis. Although the salt treatment generally reduced SIR, like the other response variables, there was no consistent, statistically significant effect across the sites and sample dates.

**Fig 5:**
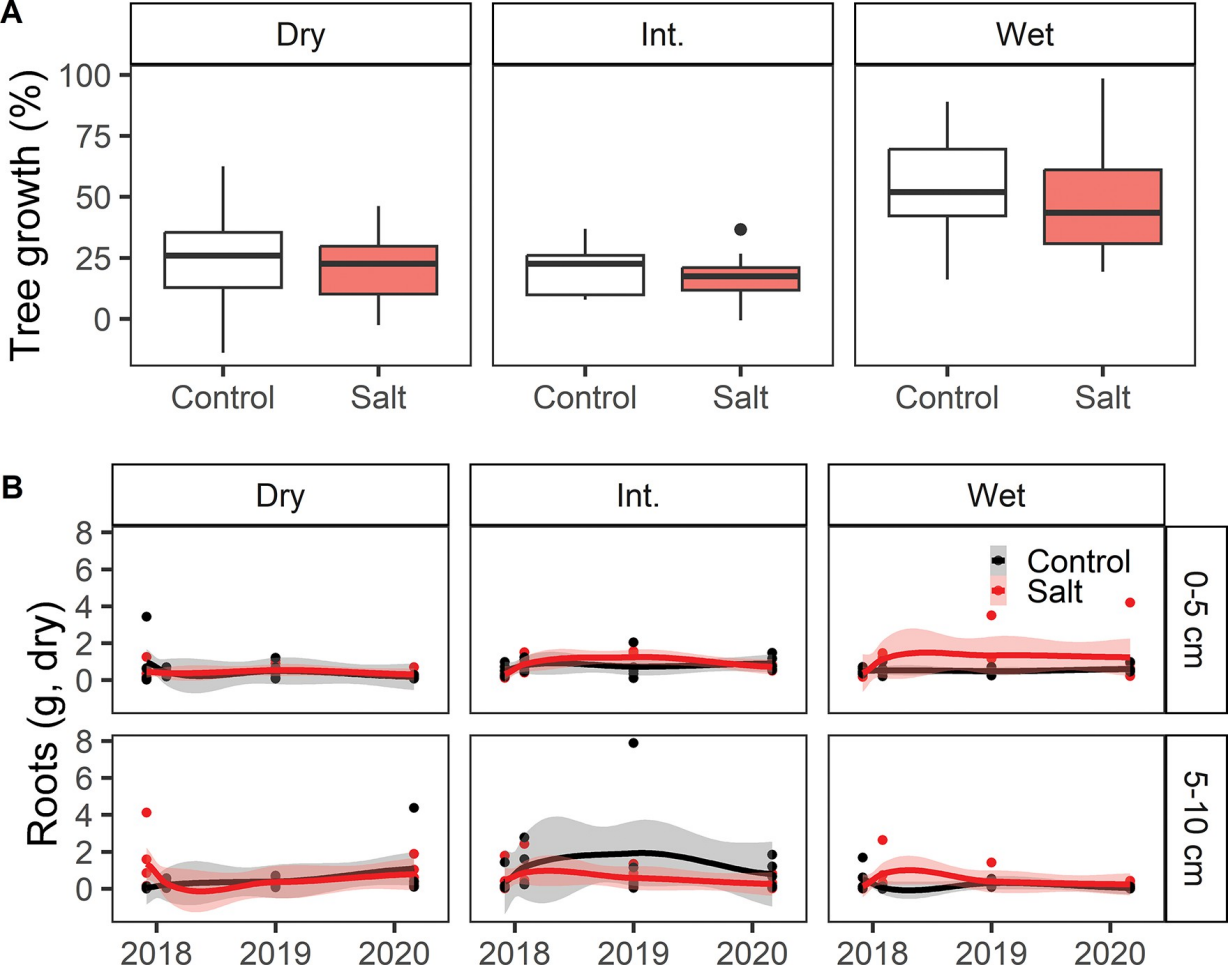
Vegetation responses to salt addition treatment. (A) Growth (%) of trees in control versus salt treatment plots from November 2015 to January 2021, averaged across all species within a plot. (B) Dry root biomass (g) from soil cores for each sampling date and site. Trend lines depict Loess-smoothed local polynomial regression and standard error shading.

We observed no significant effect of the salt treatment on either tree growth or root biomass over the duration of the experiment (Fig 5). Tree growth at the species level did not show any significant effect of the salt treatment, although the sample size was relatively small for most species (S4 Fig).

## Discussion

The outcome of this experiment defied our expectations of how salinization could impact a restored coastal forested wetland. The vegetation and soil carbon stocks proved surprisingly resistant to four-years of salt additions and where we did see treatment effects, they were highly variable across space and time. Our experimental results emphasize the importance of contextual factors, including cite hydrology and soil characteristics, in driving heterogeneous responses to salinity, even within a small geographic area with shared site history. Several previous studies from this field site have amply demonstrated the importance of hydrologic context for understanding the impact of salinization on biogeochemical processes [20, 25, 49]. Here we expand upon this understanding, to show that soil characteristics, particularly pH, base saturation and exchangeable acidity alter the magnitude and the ionic composition of our experimental salt addition.

Counter to our expectations, the salt addition did not raise the alkalinity of the soils, rather, we observed a small degree of acidification in general across the sampling dates. Similar to the findings of Ury et al. [13], the addition of base cations may stimulate the release of exchangeable acidity. Furthermore, the observed variation in exchangeable acidity across sites helps explain the variable effect of the salt treatment on the resulting pH. This observation demonstrates the importance of soil characteristics and matrix effects in governing trajectories of global change.

Our experiment unintentionally demonstrated the importance of shifting baselines with respect to environmental change. Though our experimental site did not naturally become saltier in response to sea level rise, it did become more acidic, likely in response to organic acid build up as the system recovered from pre-restoration agricultural land use. Climate change, land use legacies, agricultural inputs, and ecosystem change following both disturbance and restoration can all play a role in altering the biogeochemical status of a landscape [50–53]. Ecosystem recovery may be actively altering the baseline conditions within our experiment and interacting with the effect of our salt treatment. Organic acid accumulation may render soils more resistant to salinization, by providing additional ion binding capacity and providing a buffer against direct salt stress to organisms [18, 23, 43].

The carbon cycling processes of this forested freshwater wetland proved surprisingly resistant to four years of marine salt additions. Smaller scale, manipulative experiments conducted previously at these field sites document reduced DOC concentrations, SIR, carbon mineralization, and soil CO_2_ flux in response to salinization [20, 25, 49]. In this much larger, multi-year study, there were few consistent impacts of salinization on ecosystem carbon stocks or fluxes. It was only on our final sampling date that we detected a significant decline in soil carbon lability (both DOC and phenolic concentrations), and a significant reduction in carbon mineralization in response to the salt treatment as we had hypothesized. We did not, however, see the anticipated decrease in overall carbon stocks, which may be due to the great size of this carbon pool. Remarkably, there were no statistically significant differences in vegetation responses (tree growth and root biomass) despite a previous study by Middleton and David [54] finding significant declines in the root biomass of *Taxodium distichum* following a natural salinization event. In the case of Middleton and David [54], the salinity exceeded that of our study (reaching over 5 ppt), additionally the resistance we observed may be a testament to the vigor of a relatively young, healthy system.

The impact of our experimental salt additions was subtle, in part because the wetland did not retain as much salt as we anticipated it would. Our mode of salt addition generated a realistic imitation of how natural salinization occurs in this landscape–salinity spikes during dry periods due to evaporative concentration of salts brought it through channels and storm surge [25]. Nevertheless, treatment efficacy was undoubtedly affected by salt leaching out of treatment plots via lateral flow. Both precipitation and water level in the experimental plots were higher than average conditions for this region, reducing the intended potency of our salt treatment.

A primary motivation for conducting this large-scale and multi-year field salinization experiment arose from divergent responses between laboratory and field experiments both across this site and elsewhere in the literature. Experimental salinization suppressed CO_2_ flux in two prior incubation experiments conducted with intact soil columns from this field site [20, 49]. Yet in a small-scale field experiment, salinization had no effect on soil CO_2_ fluxes [49]. One hypothesis is that the difference in salinization impacts arises because soil-only incubations remove the potential plant-soil feedbacks and eliminate new inputs of photosynthate. In the Helton et al. [49] experiments, salinization treatment plots were too small to impact trees, but our large-scale field experiment was designed to affect both vegetation and soil heterotrophs. Previous work also shows that salt effects are highly contingent on hydrology in this region [20, 25, 49] and that responses to elevated salinity are transient [22, 26].

We observed salt suppression of carbon mineralization and a reduction in soluble organic carbon only during the final year of our experiment. We predicted that carbon solubility and lability would decrease in response to the salt addition due to flocculation caused by increased ionic strength and cation bridging [55–57]. Our results only matched these predictions on our final sampling date, and we are unable to disentangle the effect of salts compounding over time, versus an artifact of the altered sampling schedule imposed by Covid19 that affected the 2020 field season. This raises questions about the duration of responses to salinization following experimental treatments or natural saltwater intrusion events, which should be the focus of further investigation.

We observed responses to salinity that varied over space and time. Temporal variations in responses to salinization have often been observed in prior studies, particularly those conducted over long time periods. In a field addition experiment conducted in South Carolina by Neubauer [26], salt suppressed CO_2_ fluxes from wetland plots, but only in the second year of this two year study. In a year-long soil core experiment, Weston and others [22] found salt stimulated the production of CO_2_, but only during the first six months of treatments. Both site and salt treatment level are also major sources of variability driving carbon flux responses to salinity. In a study using soil from three tidal wetlands in Georgia, the magnitude of the effect of salinity on soil respiration was variable across all three sites. At the low salt dose (2 ppt) there was consistently a positive relationship between salinity and CO_2_ flux, but at the higher dose (5 ppt), the treatment only stimulated CO_2_ production at one of three sites while the other two were not significantly different from the control treatment [58].

Variability in carbon cycling responses to salt addition experiments is reflected throughout the literature. Several previous studies have reported increases in microbial respiration due to increased salinity (Table 3; [21–24, 58]). Unlike the work in this study, these experiments were short-term, and conducted as anoxic soil slurry assays in a lab setting. Longer term field studies and those conducted with intact soil cores more often showed salt suppression of CO_2_ production [19, 20, 24, 49, 59, 60], or no significant relationship [61, 62]. The only anoxic soil slurry study to demonstrate salt suppression of CO_2_ production was by Wen et al. [18] using soils from North Carolina and is consistent with other studies from this region. A key commonality between these studies may be local soil edaphic factors, in particular high iron content that buffers soils from hydrogen sulfide toxicity [16].

**Table 3:** Summary of experimental salt addition studies and effects on CO_2_ flux and extractable DOC.

Salt effect^1^ on CO_2_	Salt effect on DOC	Salinity, ppt^2^ *(effective)*	Method	Anaerobic	Duration	pH	Ecosystem	Location	Reference
↑ 24% ↑ 39%	-- --	2 5	Soil slurry (1:1)^3^	✓	2 days	--	Tidal freshwater marsh	SC	[24]
↑ Variable	-- --	2 5	Soil slurry (1:2)	✓	5 days	--	Tidal freshwater forest	GA	[58]
↑ 20% ↑ 29% ↑ 32%	-- -- --	3.5 14 35	Soil slurry (1:1)	✓	2 weeks	6.8	Freshwater wetland	FL	[21]
↑ ^4^	N.S.	10	Flow-through cores	✓	3 weeks	7.1	Unvegetated, intertidal freshwater creek bank	GA	[23]
↑ ^5^	N.S.	5	Intact soil cores	(✓)^6^	1 year	--	Tidal freshwater marsh	NJ	[22]
N.S.	↑	15	Intact peat monoliths in outdoor mesocosms		6 weeks	7.8–8.0	Mangrove peat	FL	[61]
N.S. N.S.	N.S. N.S.	1 (NaCl) 5 (NaCl)	Soil slurry (1:3)		60 days	5.0^7^	Forested wetland	SC	[62]
↓ ^8^	--	10.2 *(2–5)*	Field addition measured in situ		20 months		Tidal freshwater marsh	SC	[26]
N.S. ↓	--	18 18 (-SO_4_^2-^)	Intact soil cores, measured directly		7 weeks		Tidal *Typha* wetland	CT	[19]
↓ ↓	-- --	18 18 (-SO_4_^2-^)	Intact soil cores → bottle assay						
↓ ↓	--	8 8 (-SO_4_^2-^)	Soil slurry (1:2)		11 weeks	4.35–6.73	Forested wetland	NC	[18]
↓ ↓	--	8 8 (-SO_4_^2-^))	Soil slurry (1:2)	✓					
↓ 76%	↓ 49%	5	Intact soil cores measured directly		16 weeks	--	Forested wetland	NC	[20]
↓ ^9^ ↓ ^9^	-- --	5 5 (-SO_4_^2-^)	Intact soil cores measured directly		16 weeks	--	Forested wetland	NC	[49]
N.S. N.S.	-- --	4 10	Field addition measured in situ		15 weeks	3–4			
↓ ↓	-- --	4 10	Field addition → soil slurry (1:2)						
↓49%	↓	(2.1)	Field addition → soil slurry	✓	3.5 years	--	Tidal freshwater marsh	SC	[24]
↓^10^	↓^10^	(1–3)	Field addition → bottle assay		4 years	4–6	Forested wetland	NC	This study

^1^Arrows indicate the direction of the influence of salt treatment compared to freshwater controls; magnitude given for sources that reported an average percent change between salt treatment and control group

^2^Target salinity treatment and the effective, or measured salinity shown in parentheses, where reported

^3^Soil to water/treatment solution ratio within the slurry

^4^Measured as DIC

^5^Significant effect of the treatment only the first six months of the experiment;^6^Cores were house in an artificial tide chamber on a 6-hour inundation cycle

^7^Reported increases in pH over the duration of the experiment

^8^Year two only (year one N.S.)

^9^For intermittent flooding case only, permanently flooded cores showed no significant effect

^10^Significant effect of salt only in year four of the experiment. (-- = Not measured or not reported; N.S. = no significant effect of treatment; -SO_4_^2-^ = Artificial seawater without sulfate)

We examined the body of literature on carbon flux responses to salt addition experiments in search of patterns that drive divergent outcomes. Site edaphic factors are likely an important source of variability, however lack of consistency in reporting edaphic factors makes it difficult to draw definitive conclusions [48]. In studies that measure soil organic matter content, extractable dissolved organic carbon, and other measures of carbon quality, these metrics are often reported as cofactors mediating CO_2_ fluxes, rather than responses to salinity themselves [61]. Our results demonstrate suppression of bulk DOC and aromaticity in soil carbon due to salinity treatments. More work is needed to understand the importance of this pathway in controlling soil respiration relative to the direct effects of salt on microbial function [12].

Disentangling the effects of salinization on wetland carbon cycling across time and space will require a concerted effort to standardize methods of study going forward. Consistency of methods will help facilitate comparison across studies from different locations. Edaphic factors, particularly pH, soil iron content, and ion exchange capacity, are important covariates that modify the effect of salinization on carbon cycling. These factors should be reported along with salinity in order to understand these interaction effects. Another revelation from this experiment is the potential for discrepancies between target salt treatment level and salinity achieved, even across replicated plots. Future investigations should report both target and realized values salt levels, as well as any long-term changes to initial soil conditions such as pH or soil carbon content or quality. Finally, it is important that the results of short-term lab experiments be considered in concert with longer field studies. While lab experiments and short-term field studies are often preferred due to cost and feasibility, our study highlights the importance of multi-year, large-scale field experiments for uncovering environmental controls on ecosystem processes. Armed with a more nuanced understanding of these interactions, we can better constrain models of these processes and improve future predictions of changes to coastal carbon stocks. Only by challenging our phenomenological observations with environmental complexity do we further our understanding of how ecosystems are responding to multiple drivers of change.

## Conclusions

Ecological responses to a large-scale salt addition in a coastal freshwater wetland were highly variable across space and time. We observed suppression of soil respiration and a reduction in soil carbon lability; however, these effects were only statistically significant during the fourth year of the experiment. There was little accumulation of the applied salts within the experimental plots, and no statistically significant effects on vegetation (tree growth and root biomass) were observed. Both the heterogeneous response to salt treatments as well as the resistance of restored wetlands to salt stress should be the topic of further research efforts. Understanding the impacts of salinization on coastal carbon cycling has broader implications for our ability to accurately model and predict changes in coastal ecosystem function in the face of climate change.

## Supporting information

S1 Fig Experimental timeline. Timeline of salt additions (black triangle) beginning in Fall 2016 through summer 2020. Conductivity data from loggers located in sampling wells illustrates the effect of the salt additions on pore water salinity. This plot shows data from the wet site only and gaps indicate periods when groundwater level dropped below sensor depth or other sensor failure.
(PNG)

S2 Fig Soil carbon responses to salt treatment. (A) Soil carbon mineralization rate per gram dry soil, (B) substrate induced respiration (SIR) on a per gram carbon basis, and (C) SIR on a per gram dry soil basis shown for each sampling date, site, and depth. Trend lines depict Loess-smoothed local polynomial regression and standard error shading.
(TIF)

S3 Fig Soil phenolic compound content response to salt treatment. (A) Concentration of water extractable phenolic compounds and (B) phenolic compounds on a per mass of dissolved organic carbon basis (DOC) for each sampling date, site, and depth. Trend lines depict Loess-smoothed local polynomial regression and standard error shading.
(TIF)

S4 Fig Tree growth response to salt treatment by species. Tree growth (%) in control versus salt treatment plots from November 2015 to January 2021 by species (species with fewer than 3 occurrences excluded).
(TIF)

S1 Table Salt addition and sampling schedule. (DOCX)

S2 Table Statistical summary. (DOCX)
