# Supplementary figures and images for: Restored forested wetland surprisingly resistant to experimental salinization
Source: PLoS One. 2023 Dec 21;18(12):e0296128. doi: 10.1371/journal.pone.0296128 (PMC10734931; doi:10.1371/journal.pone.0296128)

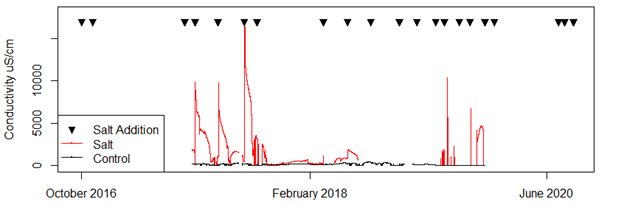

Supplement: S1 Fig — Timeline of salt additions (black triangle) beginning in Fall 2016 through summer 2020. Conductivity data from loggers located in sampling wells illustrates the effect of the salt additions on pore water salinity. This plot shows data from the wet site only and gaps indicate periods when groundwater level dropped below sensor depth or other sensor failure. (PNG) [file pone.0296128.s001.png]

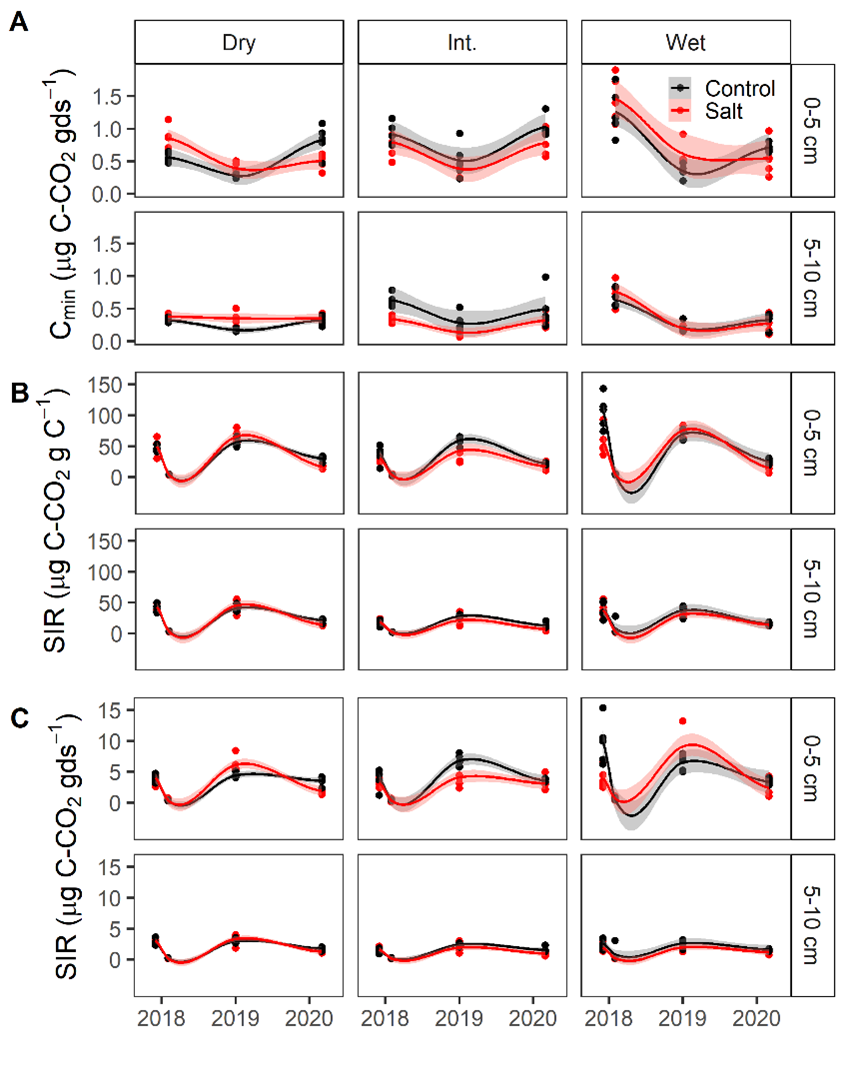

Supplement: S2 Fig — (A) Soil carbon mineralization rate per gram dry soil, (B) substrate induced respiration (SIR) on a per gram carbon basis, and (C) SIR on a per gram dry soil basis shown for each sampling date, site, and depth. Trend lines depict Loess-smoothed local polynomial regression and standard error shading. (TIF) [file pone.0296128.s002.tif]

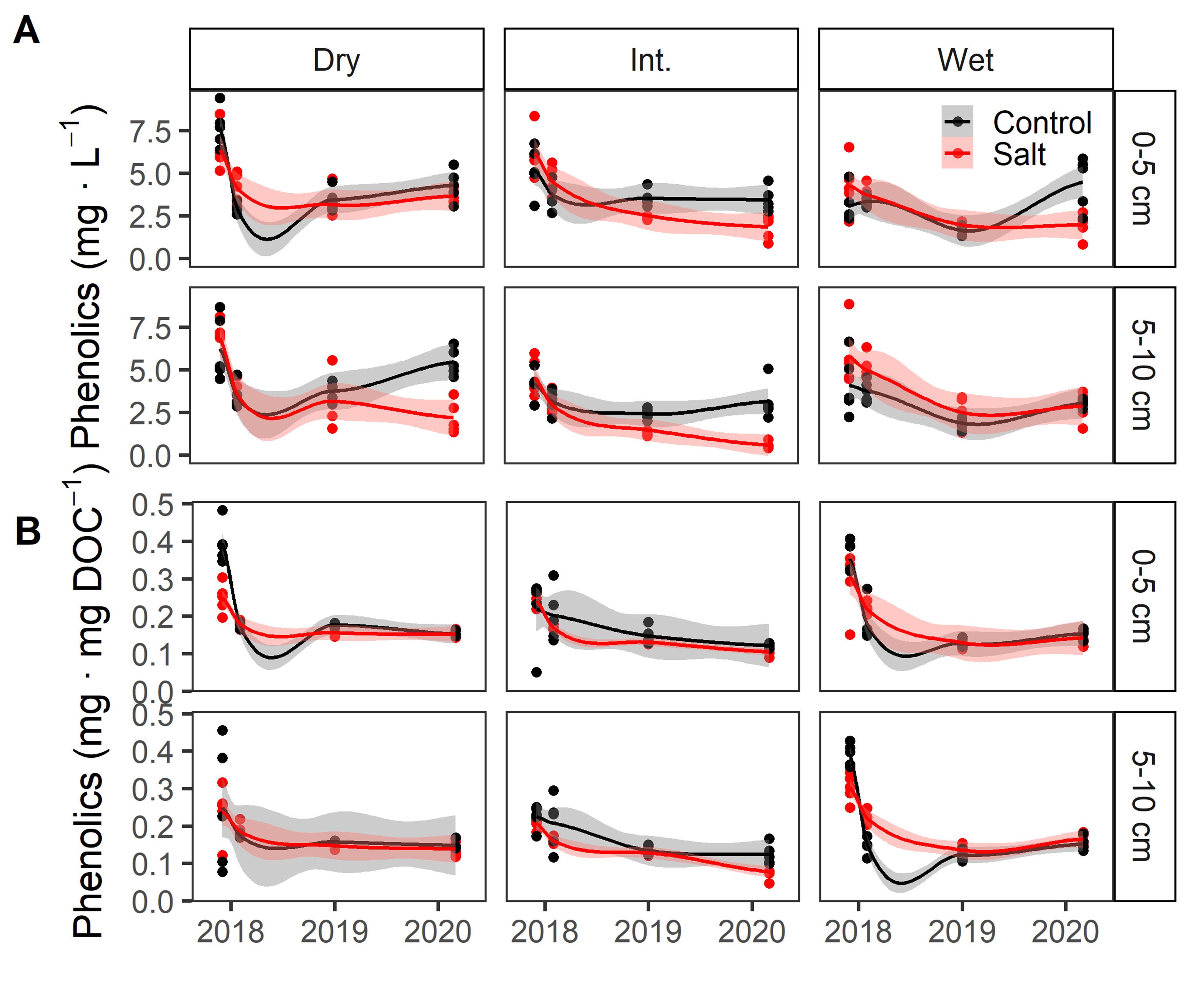

Supplement: S3 Fig — (A) Concentration of water extractable phenolic compounds and (B) phenolic compounds on a per mass of dissolved organic carbon basis (DOC) for each sampling date, site, and depth. Trend lines depict Loess-smoothed local polynomial regression and standard error shading. (TIF) [file pone.0296128.s003.tif]

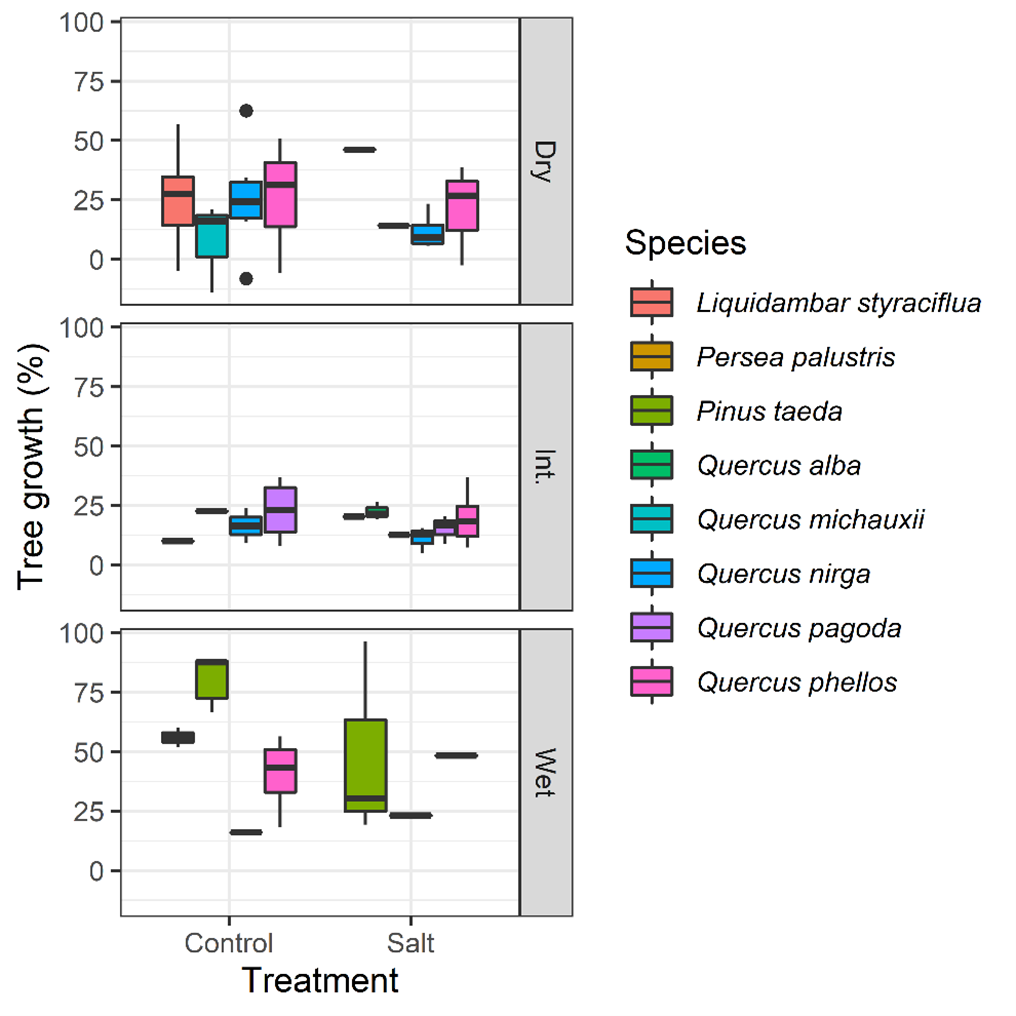

Supplement: S4 Fig — Tree growth (%) in control versus salt treatment plots from November 2015 to January 2021 by species (species with fewer than 3 occurrences excluded). (TIF) [file pone.0296128.s004.tif]
