# Supplementary material for: Restored forested wetland surprisingly resistant to experimental salinization
Source: PLoS One. 2023 Dec 21;18(12):e0296128. doi: 10.1371/journal.pone.0296128 (PMC10734931; doi:10.1371/journal.pone.0296128)
Supplement: S1 Table — (DOCX) [file pone.0296128.s005.docx]

**S1 Table. Salt addition and sampling schedule**

| Year | Date | Activity |
| --- | --- | --- |
| 2015 | November | Sites established; trees measured |
| 2016 | October 5 | Salt additions begin |
|  | November 3 | Salt addition |
| 2017 | March 7 | Salt addition |
|  | May 4 | Salt addition |
|  | July 5 | Salt addition |
|  | October 15 | Salt addition |
| 2018 | April 9 | Salt addition |
|  | May 10 | **First soil sampling** |
|  | June 11 | Salt addition |
|  | July 10 | **Second soil sampling** |
|  | August 8 | Salt addition |
|  | October 27 | Salt addition |
| 2019 | February 28 | Salt addition |
|  | March 29 | Salt addition |
|  | April 30 | Salt addition |
|  | May 21 | Salt addition |
|  | June 20 | **Third soil sampling** |
|  | June 30 | Salt addition |
|  | July 22 | Salt addition |
|  | August 19 | Salt addition |
|  | September 28 | Salt addition |
|  | October 12 | Salt addition |
| 2020 | June 26 | Salt addition |
|  | July 13 | Salt addition |
|  | August 5 | Salt addition |
|  | August 8 | **Fourth soil sampling** |
| 2021 | January 16 | Trees measured |
