# Supplementary material for: Restored forested wetland surprisingly resistant to experimental salinization
Source: PLoS One. 2023 Dec 21;18(12):e0296128. doi: 10.1371/journal.pone.0296128 (PMC10734931; doi:10.1371/journal.pone.0296128)
Supplement: S2 Table — (DOCX) [file pone.0296128.s006.docx]

**S2 Table. Statistical summary.**

|  | |  | Site 1 | | Site 3 | | Site 5 | |
| --- | --- | --- | --- | --- | --- | --- | --- | --- |
|  |  | Depth | Control | Salt | Control | Salt | Control | Salt |
| Cl^-^ (μg/gds) | | |  |  |  |  |  |  |
|  | May 10th, 2018 | (0-5)  (5-10) | 64.3 (10.27) 59.73 (10.97) | 532.3 (265.82) 685.0 (367.37) | **105.25 (6.68) 67.13 (12.3)** | **975.71 (45.2) 978.51 (79.57)** | **123.92 (23.98) 58.36 (11.96)** | **419.8 (28.15) 467.82 (74.2)** |
|  | July 10th, 2018 | (0-5)  (5-10) | **63.63 (9.83) 47.24 (5.11)** | **1444.7 (371) 1148.6 (273)** | **511.31 (104.4) 532.54 (50.37)** | **2019.2 (259.9) 1756.7 (184.2)** | **572.26 (36.82)** 476.5 (17.49) | **910.45 (98.9)** 575.87 (227.8) |
|  | June 20th, 2019 | (0-5)  (5-10) | **6.2 (0.82) 7.44 (1.38)** | **616.07 (99.8) 511.68 (82.1)** | **28.13 (8.48) 5.21 (0.56)** | **306.1 (94.34) 336.93 (99.05)** | **31.04 (5.84) 20.73 (9.15)** | **328.17 (64.9) 261.37 (17.1)** |
|  | August 8th, 2020 | (0-5)  (5-10) | **10.02 (1.44) 7.23 (1.08)** | **170.5 (35.5) 601.1 (199.8)** | **35.31 (14.02) 22.78 (4.38)** | **2571.8 (1033) 2848.6 (1001)** | 11.43 (2.08) **9.37 (1.02)** | 972.8 (455.8) **273.16 (81.1)** |
| SO_4_^2-^ (μg/gds) | |  |  |  |  |  |  |  |
|  | May 10^th^, 2018 | (0-5)  (5-10) | 117.84 (17.17) 137.84 (27.87) | 298.47 (81.04) 256.28 (74.02) | 136.46 (26.37) **76.78 (2.34)** | 238.59 (38.39) **160.54 (33.09)** | 109.6 (25.83) 68.8 (9.63) | 148.97 (9.2) 67.67 (6.62) |
|  | July 10^th^, 2018 | (0-5)  (5-10) | **76.35 (12.29) 57.01 (10.61)** | **323.21 (72.2) 380.24 (95.4)** | 277.31 (63.55) 143.26 (43.37) | 379.51 (82.17) 268.4 (52.63) | 166.91 (26.13) 121.46 (15.84) | 177.55 (27.41) 86.14 (13.02) |
|  | June 20^th^, 2019 | (0-5)  (5-10) | **11.17 (0.18) 13.81 (1.16)** | **132.39 (15.0) 109.23 (10.3)** | **20.8 (4.29) 13.8 (1.13)** | **79.98 (18.24) 70.54 (13.85)** | **35.08 (4.9) 30.52 (11.77)** | **106.25 (23.7) 87.31 (17.1)** |
|  | August 8^th^, 2020 | (0-5)  (5-10) | **10.09 (0.62) 8.94 (0.51)** | **78.27 (18.34) 141.75 (38.4)** | **23.48 (3.14) 21.61 (2.6)** | **441.56 (167.7) 400.6 (141.28)** | 12.75 (0.91) 11.57 (0.63) | 134.33 (52.75) 38.08 (12.43) |
| Na^+^ (μg/gds) | | |  |  |  |  |  |  |
|  | May 10^th^, 2018 | (0-5)  (5-10) | 522.48 (58.04) 490.9 (12.45) | 966.07 (184.1) 949.34 (224.5) | **618.85 (43.08) 567.59 (13.59)** | **1232.53 (30.4) 1187.1 (44.4)** | **492.22 (115.7) 456.23 (28.9)** | **879.03 (19.3) 789.87 (52.5)** |
|  | July 10^th^, 2018 | (0-5)  (5-10) | **160.14 (11.73) 168.98 (3.24)** | **1264.7 (265) 1033.9 (197)** | **399.14 (53.67) 739.54 (40.96)** | **1408.66(161.8) 1482.15(114.1)** | **731.61 (24.17)** 667.83 (6.97) | **1219.7 (77.5)** 784.95 (93.1) |
|  | June 20^th^, 2019 | (0-5)  (5-10) | **34.1 (2.54) 35.55 (2.26)** | **444.82 (57.9) 347.53 (37.9)** | **57.57 (6.37) 47.17 (2.75)** | **241.72 (56.2) 238.83 (57.26)** | **56.74 (4.39) 44.57 (5.57)** | **261.72 (31.2) 187.56 (12.2)** |
|  | August 8^th^, 2020 | (0-5)  (5-10) | **14.5 (0.89) 12.18 (0.23)** | **167.05 (25.0) 416.7 (121.3)** | **44.3 (5.96) 32.23 (1.83)** | **1573.71 (594) 1680.75 (595)** | **22.33 (1.55) 17.02 (1.12)** | **615.09 (240) 212.53 (39.2)** |
| Water extractable soil ion content (Cl, SO_4_, Na) by site, treatment, depth, and date, reporting mean (standard error, n = 5). Bolding indicates a statistically significant difference between salt and control (one-way ANOVA, α = 0.05). | | | | | | | | |

**S2 Table (continued). Statistical summary.**

|  | |  | Site 1 | | Site 3 | | Site 5 | |
| --- | --- | --- | --- | --- | --- | --- | --- | --- |
|  |  | Depth | Control | Salt | Control | Salt | Control | Salt |
| K^+^ (μg/gds) | | |  |  |  |  |  |  |
|  | May 10th, 2018 | (0-5)  (5-10) | 43.89 (2.7) **25.96 (4.87)** | 67.52 (11.9) **72.45 (15.67)** | 70.53 (9.99) **30.24 (5.34)** | 83.4 (5.8) **60.06 (9.69)** | 31.51 (6.14) 7.99 (1.38) | 58.24 (12.15) 27.67 (8.64) |
|  | July 10th, 2018 | (0-5)  (5-10) | **16.55 (3.29) 11.14 (0.51)** | **86.57 (13.81) 63.07 (11.07)** | **80.18 (12.61) 44.45 (5.97)** | **123.63 (10.05) 115.17 (8.08)** | **27.19 (1.26) 31.98 (6.63)** | **64.54 (6.36) 58.99 (5.72)** |
|  | June 20th, 2019 | (0-5)  (5-10) | **2.04 (0.22) 1.72 (0.07)** | **28.88 (3.05) 19.69 (1.14)** | 12.11 (1.75) 4.47 (0.62) | 15.93 (3.5) 10.07 (2.39) | **2.23 (0.36) 1.6 (0.19)** | **10.39 (2.43) 4.88 (0.77)** |
|  | August 8th, 2020 | (0-5)  (5-10) | **3.82 (0.84) 1.68 (0.19)** | **16.06 (2.74) 27.67 (7.03)** | **11.74 (2.48) 5.1 (0.57)** | **76.31 (25.19) 49.42 (15.91)** | **2.59 (0.27) 1.28 (0.12)** | **30.17 (11.03) 7.9 (1.17)** |
| Ca^2+^ (μg/gds) | |  |  |  |  |  |  |  |
|  | May 10^th^, 2018 | (0-5)  (5-10) | **194.95 (25.58) 484 (159.33)** | **18.98 (5.94) 52.47 (23.09)** | 4.04 (4.04) 0.3 (0.29) | b.d.  b.d. | **260.51 (12.32) 129.78 (12.72)** | **204.47 (15.1) 195.4 (17.48)** |
|  | July 10^th^, 2018 | (0-5)  (5-10) | **319.28 (7.51) 428.12 (36.62)** | **91.42 (21.36) 112.52 (15.1)** | 111.44 (13.36) **170.61 (12.12)** | 84.17 (9.57) **92.39 (4.31)** | **555.71 (19.21) 541.77 (21.71)** | **341.64 (24.5) 401.46 (31.5)** |
|  | June 20^th^, 2019 | (0-5)  (5-10) | 44.88 (6.26) **62.14 (4.48)** | 28.6 (5.12) **38.67 (7.28)** | 8.48 (1.63) **6.97 (0.47)** | 11.25 (1.45) **17.88 (1.61)** | 39.73 (1.92) **46.81 (4.48)** | 57.38 (14.27) **60.98 (3.84)** |
|  | August 8^th^, 2020 | (0-5)  (5-10) | **26.37 (1.11)** 25.98 (1.03) | **5.65 (0.63)** 28.34 (8.53) | 6.99 (1.15) **9.22 (1.03)** | 37.29 (16.12) **46.38 (12.27)** | 32.76 (3.95) 31.24 (1.11) | 76.13 (29.12) 35.19 (8.13) |
| Mg^2+^ (μg/gds) | | |  |  |  |  |  |  |
|  | May 10^th^, 2018 | (0-5)  (5-10) | **15.9 (1.5)** 49.59 (21.19) | **6.37 (2.67)** 11.44 (4.2) | **0.67 (0.45) 0 (0)** | **8.56 (2.26) 12.79 (4.92)** | 61.11 (5.06) **18.93 (4.44)** | 66.07 (10.02) **59.42 (5.96)** |
|  | July 10^th^, 2018 | (0-5)  (5-10) | 31.91 (1.53) 31.5 (5.24) | 40.5 (7.61) 32.27 (9.55) | **46.65 (7.43)** 11.01 (3.9) | **99.99 (13.78)** 36.69 (11.81) | 86.27 (5.35) 66.43 (3.34) | 84.23 (7.14) 70.54 (12.73) |
|  | June 20^th^, 2019 | (0-5)  (5-10) | **3.42 (0.44) 3.96 (0.29)** | **25.73 (6.19) 27.13 (6.5)** | **3.44 (0.98) 0.04 (0)** | **24.86 (7.42) 22.92 (7.35)** | 5.81 (0.54) **5.51 (1.71)** | 24.82 (8.46) **20.55 (2.85)** |
|  | August 8^th^, 2020 | (0-5)  (5-10) | **1.64 (0.18) 0.66 (0.2)** | **6.59 (1.83) 35.58 (14.12)** | **0.27 (0.18) 0 (0)** | **172.62 (73.97) 174.89 (41.81)** | 3.41 (0.42) 2.66 (0.24) | 55.16 (32.67) 11.79 (4.86) |
| Water extractable soil ion content (K, Ca, Mg) by site, treatment, depth, and date, reporting mean (standard error, n = 5). Bolding indicates a statistically significant difference between salt and control (one-way ANOVA, α = 0.05). | | | | | | | | |

**S2 Table (continued). Statistical summary.**

|  | |  | Site 1 | | Site 3 | | Site 5 | |
| --- | --- | --- | --- | --- | --- | --- | --- | --- |
|  |  | Depth | Control | Salt | Control | Salt | Control | Salt |
| pH | | |  |  |  |  |  |  |
|  | May 10th, 2018 | (0-5)  (5-10) | 6.06 (0.05) 6.36 (0.05) | 5.66 (0.19) 5.82 (0.24) | 4.66 (0.07) 4.98 (0.04) | 4.78 (0.04) 4.88 (0.09) | **6.24 (0.09)** 6.66 (0.02) | **6.62 (0.04)** 6.6 (0.05) |
|  | July 10th, 2018 | (0-5)  (5-10) | **5.68 (0.09) 5.92 (0.05)** | **5.08 (0.12) 5.28 (0.09)** | 4.7 (0.1) 5.2 (0.1) | 4.84 (0.07) 5.06 (0.07) | 6.3 (0.03) 6.36 (0.04) | 6.32 (0.02) 6.48 (0.06) |
|  | June 20th, 2019 | (0-5)  (5-10) | **4.88 (0.11) 5.25 (0.04)** | **4.25 (0.07) 4.39 (0.07)** | 4 (0.14) **4.58 (0.04)** | 3.94 (0.08) **4.2 (0.07)** | 5.51 (0.12) **5.9 (0.05)** | 5.49 (0.01) **5.5 (0.04)** |
|  | August 8th, 2020 | (0-5)  (5-10) | 4.67 (0.08) **4.9 (0.04)** | 4.48 (0.06) **4.4 (0.07)** | 3.62 (0.07) **3.82 (0.05)** | 3.6 (0.05) **3.62 (0.04)** | 5.16 (0.05) 5.41 (0.05) | 5.33 (0.07) 5.51 (0.1) |
| Soil moisture (%) | |  |  |  |  |  |  |  |
|  | May 10^th^, 2018 | (0-5)  (5-10) | 26.82 (0.39) **24.8 (0.49)** | 25.16 (1.29) **22.69 (0.45)** | 32.26 (0.98) 30.14 (0.62) | 33.02 (0.59) 30.56 (0.39) | 36.78 (2.16) 28.55 (0.48) | 32 (2.11) 25.63 (1.18) |
|  | July 10^th^, 2018 | (0-5)  (5-10) | **13.1 (0.56)** 12.28 (0.69) | **18.55 (1.21)** 14.42 (0.92) | 31.01 (1.06) 27.75 (0.86) | 30.76 (0.53) 27.45 (0.29) | 32.73 (0.81) 27.59 (0.51) | 38.93 (2.76) 28.63 (1.27) |
|  | June 20^th^, 2019 | (0-5)  (5-10) | **20.97 (0.69)** 20.38 (0.47) | **24.76 (0.56)** 21.04 (0.51) | 26.4 (1.69) 25.62 (0.49) | 30.17 (0.35) 26.21 (0.1) | **22.62 (1.51)** 20.58 (1.08) | **37.83 (1.89)** 24.2 (1.28) |
|  | August 8^th^, 2020 | (0-5)  (5-10) | 29.8 (0.81) 24 (0.55) | 29.56 (0.67) 27.38 (1.49) | 32.99 (0.65) **29.15 (0.35)** | 34.64 (0.69) **30.8 (0.44)** | 34.62 (2.46) 28.97 (0.56) | 39.73 (2.01) 28.48 (2.61) |
| Bulk Density (g/cm^3^) | | |  |  |  |  |  |  |
|  | May 10^th^, 2018 | (0-5)  (5-10) | 1.36 (0.05) 1.55 (0.04) | 1.21 (0.05) 1.46 (0.04) | 1.41 (0.03) 1.49 (0.02) | 1.3 (0.04) 1.48 (0.02) | **1.31 (0.05)** 1.54 (0.01) | **1.56 (0.1)** 1.6 (0.03) |
|  | July 10^th^, 2018 | (0-5)  (5-10) | 1.08 (0.03) 1.38 (0.03) | 1.02 (0.05) 1.36 (0.02) | 1.38 (0.02) **1.47 (0.03)** | 1.45 (0.04) **1.53 (0.04)** | 1.23 (0.03) 1.52 (0.02) | 1.31 (0.03) 1.48 (0.05) |
|  | June 20^th^, 2019 | (0-5)  (5-10) | **1.27 (0.06)** 1.42 (0.06) | **1.15 (0.03)** 1.45 (0.07) | **1.14 (0.07)** 1.49 (0.03) | **1.27 (0.04)** 1.45 (0.02) | 0.95 (0.05) 1.3 (0.06) | 0.98 (0.09) 1.49 (0.07) |
|  | August 8^th^, 2020 | (0-5)  (5-10) | **1.48 (0.03) 1.55 (0.02)** | **1.49 (0.03) 1.44 (0.05)** | 1.43 (0.02) **1.5 (0.02)** | 1.42 (0.02) **1.45 (0.03)** | 1.5 (0.06) 1.52 (0.01) | 1.42 (0.08) 1.53 (0.08) |
| Soil properties (pH, soil moisture, bulk density) by site, treatment, depth, and date, reporting mean (standard error, n = 5). Bolding indicates a statistically significant difference between salt and control (one-way ANOVA, α = 0.05). | | | | | | | | |

**S2 Table (continued). Statistical summary.**

|  | |  | Site 1 | | Site 3 | | Site 5 | |
| --- | --- | --- | --- | --- | --- | --- | --- | --- |
|  | | Depth | Control | Salt | Control | Salt | Control | Salt |
| Roots (g) | |  |  |  |  |  |  |  |
|  | May 10^th^, 2018 | (0-5)  (5-10) | 0.97 (0.63) 0.05 (0.02) | 0.45 (0.21) 1.35 (0.75) | 0.52 (0.15) 0.37 (0.26) | 0.33 (0.11) 0.49 (0.33) | 0.51 (0.07) 0.49 (0.32) | 0.39 (0.09) 0.14 (0.11) |
|  | July 10^th^, 2018 | (0-5)  (5-10) | 0.43 (0.08) 0.22 (0.1) | 0.38 (0.03) 0.28 (0.03) | 0.81 (0.15) 1.18 (0.47) | 0.87 (0.18) 0.86 (0.41) | **0.53 (0.14)** 0.05 (0.02) | **1.13 (0.17)** 0.8 (0.47) |
|  | June 20^th^, 2019 | (0-5)  (5-10) | 0.5 (0.21) 0.39 (0.14) | 0.54 (0.19) 0.36 (0.07) | 0.73 (0.34) 1.92 (1.51) | 1.24 (0.29) 0.58 (0.22) | 0.49 (0.07) 0.31 (0.08) | 1.34 (0.55) 0.39 (0.26) |
|  | August 8^th^, 2020 | (0-5)  (5-10) | 0.18 (0.04) 1.08 (0.83) | 0.31 (0.1) 0.79 (0.31) | 0.91 (0.18) 0.79 (0.33) | 0.72 (0.13) 0.26 (0.15) | 0.6 (0.1) **0.04 (0.02)** | 1.24 (0.75) **0.24 (0.08)** |
| Tree diameter (percent growth) | | |  |  |  |  |  |  |
|  | November 2015 - January, 2021 |  | 23.6 (4.3)  n=23 | 21.3 (4.3)  n = 12 | 20.5 (3.2)  n = 11 | 16.9 (1.6)  n = 27 | 54.7 (6.0)  n = 15 | 47.3 (4.7)  n = 122 |
| Vegetation measurements by site, treatment, depth, and date, reporting mean (standard error, n = 5). Bolding indicates a statistically significant difference between salt and control (one-way ANOVA, α = 0.05). | | | | | | | | |

**S2 Table (continued). Statistical summary.**

|  | |  | Site 1 | | Site 3 | | Site 5 | |
| --- | --- | --- | --- | --- | --- | --- | --- | --- |
|  | | Depth | Control | Salt | Control | Salt | Control | Salt |
| SIR (ugC-CO2/hr/goc) | | |  |  |  |  |  |  |
|  | May 10th, 2018 | (0-5)  (5-10) | 46.87 (2.47) 40.09 (2.88) | 49.33 (5.83) 42.8 (2.95) | 36.34 (6.22) 15.17 (2.34) | 30.82 (1.82) 18.04 (1.66) | **105.5 (11.98)** 37.7 (5.88) | **56.7 (9.97)** 37.54 (5.58) |
|  | July 10th, 2018 | (0-5)  (5-10) | 4.22 (0.11) **3.59 (0.09)** | 3.72 (0.31) **2.87 (0.14)** | 2.85 (0.13) **1.96 (0.11)** | 2.51 (0.6) **1.5 (0.08)** | 4.5 (0.3) 7.71 (4.97) | 3.97 (0.15) 2.31 (0.31) |
|  | June 20th, 2019 | (0-5)  (5-10) | 57.01 (3.54) 40.58 (2.47) | 65.56 (4.48) 45.04 (5.69) | 59.47 (3.24) 27.49 (1) | 42.45 (8.29) 21.0 (5.45) | 69.67 (3.92) 36.74 (3.57) | 75.6 (4.29) 30.8 (3.19) |
|  | August 8th, 2020 | (0-5)  (5-10) | **29.65 (1.99) 20.71 (1.39)** | **16.71 (1.09) 13.64 (0.62)** | 21.54 (0.61) **12.26 (1.94)** | 16.94 (2.54) **6.74 (1.06)** | **25.41 (1.83)** 14.87 (1.01) | **14.69 (2.42)** 13.86 (1.04) |
| SIR (ugC-CO2/hr/gds) | | |  |  |  |  |  |  |
|  | May 10th, 2018 | (0-5)  (5-10) | 3.87 (0.27) 2.85 (0.22) | 4 (0.38) 3.1 (0.13) | 3.86 (0.7) 1.35 (0.19) | 3.25 (0.24) 1.64 (0.17) | **9.8 (1.61)** 2.58 (0.37) | **3.93 (0.71)** 1.93 (0.28) |
|  | July 10th, 2018 | (0-5)  (5-10) | 0.41 (0.02) 0.29 (0.01) | 0.45 (0.08) 0.24 (0.02) | 0.41 (0.03) **0.23 (0.02)** | 0.37 (0.1) **0.17 (0.01)** | 0.65 (0.06) 0.84 (0.56) | 0.7 (0.11) 0.22 (0.03) |
|  | June 20th, 2019 | (0-5)  (5-10) | **4.4 (0.19)** 2.96 (0.16) | **6.11 (0.6)** 3.32 (0.37) | **6.75 (0.44)** 2.41 (0.11) | **4.09 (0.69)** 1.93 (0.42) | 6.51 (0.59) 2.6 (0.26) | 9.07 (1.38) 1.98 (0.34) |
|  | August 8th, 2020 | (0-5)  (5-10) | **3.54 (0.32) 1.76 (0.12)** | **1.91 (0.16) 1.27 (0.09)** | 3.57 (0.12) **1.56 (0.2)** | 3.11 (0.53) **0.96 (0.14)** | 3.4 (0.2) 1.61 (0.09) | 2.39 (0.59) 1.2 (0.18) |
| Substrate induced respiration (SIR) by site, treatment, depth, and date, reporting mean (standard error, n = 5). Bolding indicates a statistically significant difference between salt and control (one-way ANOVA, α = 0.05). | | | | | | | | |

**S2 Table (continued). Statistical summary.**

|  | |  | Site 1 | | Site 3 | | Site 5 | |
| --- | --- | --- | --- | --- | --- | --- | --- | --- |
|  | | Depth | Control | Salt | Control | Salt | Control | Salt |
| Cmineralization (ugC-CO2/hr/goc) | | | |  |  |  |  |  |
|  | May 10th, 2018 | (0-5)  (5-10) |  |  |  |  |  |  |
|  | July 10th, 2018 | (0-5)  (5-10) | **6.73 (0.37)** 4.64 (0.22) | **8.79 (0.21)** 5.4 (0.5) | 9.25 (0.43) **7.4 (0.49)** | 7.81 (0.94) **4.03 (0.27)** | 12.82 (1.18) **8.34 (0.58)** | 14 (0.67) **11.17 (0.82)** |
|  | June 20th, 2019 | (0-5)  (5-10) | 3.6 (0.27) **2.35 (0.07)** | 4.25 (0.44) **4.65 (0.4)** | 4.41 (0.91) 3.22 (0.75) | 4.07 (0.67) 1.5 (0.33) | **3.74 (0.43)** 2.86 (0.48) | **5.11 (0.29)** 3.17 (0.15) |
|  | August 8th, 2020 | (0-5)  (5-10) | **6.9 (0.72)** 3.83 (0.4) | **4.49 (0.51)** 3.79 (0.4) | **6.09 (0.32)** 3.86 (1.12) | **4.24 (0.38)** 2.22 (0.38) | **5.34 (0.43)** 2.95 (0.41) | **3.37 (0.48)** 3.09 (0.43) |
| Cmineralization (ugC-CO2/hr/gds) | | | |  |  |  |  |  |
|  | May 10th, 2018 | (0-5)  (5-10) |  |  |  |  |  |  |
|  | July 10th, 2018 | (0-5)  (5-10) | **0.56 (0.03)** 0.32 (0.02) | **0.85 (0.08)** 0.38 (0.02) | 0.91 (0.08) **0.63 (0.04)** | 0.79 (0.12) **0.34 (0.02)** | 1.26 (0.16) 0.64 (0.06) | 1.46 (0.16) 0.76 (0.08) |
|  | June 20th, 2019 | (0-5)  (5-10) | **0.28 (0.01) 0.17 (0.01)** | **0.39 (0.04) 0.35 (0.05)** | 0.51 (0.12) 0.28 (0.07) | 0.39 (0.04) 0.14 (0.03) | 0.35 (0.04) 0.2 (0.04) | 0.49 (0.15) 0.2 (0.02) |
|  | August 8th, 2020 | (0-5)  (5-10) | **0.82 (0.1)** 0.32 (0.03) | **0.51 (0.05)** 0.35 (0.03) | 1.03 (0.07) 0.49 (0.13) | 0.78 (0.09) 0.32 (0.05) | 0.71 (0.03) 0.33 (0.05) | 0.54 (0.12) 0.27 (0.06) |
| Soil carbon mineralization rates by site, treatment, depth, and date, reporting mean (standard error, n = 5). Bolding indicates a statistically significant difference between salt and control (one-way ANOVA, α = 0.05). | | | | | | | | |

**S2 Table (continued). Statistical summary.**

|  | |  | Site 1 | | Site 3 | | Site 5 | |
| --- | --- | --- | --- | --- | --- | --- | --- | --- |
|  | | Depth | Control | Salt | Control | Salt | Control | Salt |
| DOC (mg/L) | |  |  |  |  |  |  |  |
|  | May 10th, 2018 | (0-5)  (5-10) | 19.81 (1.82) 34.03 (8.71) | 26.21 (3.41) 33.06 (8.55) | 29.81 (7.89) 18.63 (1.1) | 25.67 (2.37) 21.95 (1.91) | **8.79 (1.12) 10.71 (2.29)** | **14.09 (1.23) 19.37 (2.96)** |
|  | July 10th, 2018 | (0-5)  (5-10) | **16.67 (0.78)** 19.76 (2.04) | **22.99 (1.88)** 19.47 (2.03) | **19.39 (1.34)** 15.9 (1.12) | **27.11 (2.62)** 18.41 (1.54) | 19.59 (1.61) 24.95 (0.88) | 17.22 (1.48) 22.41 (1.19) |
|  | June 20th, 2019 | (0-5)  (5-10) | 19.45 (1.54) 23.82 (1.6) | 20.31 (2.56) 21.49 (4.45) | 24.49 (2.3) **18.25 (1.49)** | 19.45 (1.3) **11.6 (1.56)** | 13.04 (0.99) 15.34 (0.79) | 15.36 (0.52) 18.13 (2) |
|  | August 8th, 2020 | (0-5)  (5-10) | 28.24 (2.75) **36.81 (2.3)** | 24.18 (1.12) **15.32 (1.88)** | **28.43 (2.53) 25.06 (1.68)** | **17.46 (2.65) 7.76 (0.73)** | **28.83 (3.81)** 20.04 (0.61) | **13.8 (2)** 17.31 (2.02) |
| Organic matter (% LOI) | |  |  |  |  |  |  |  |
|  | May 10th, 2018 | (0-5)  (5-10) | 8.24 (0.26) 7.12 (0.26) | 8.21 (0.34) 7.32 (0.39) | 10.41 (0.47) 8.94 (0.13) | 10.49 (0.21) 9.04 (0.19) | **9.09 (0.45) 6.89 (0.11)** | **6.93 (0.3) 5.23 (0.42)** |
|  | July 10th, 2018 | (0-5)  (5-10) | 8.32 (0.25) 6.99 (0.07) | 9.61 (0.72) 7.14 (0.29) | 9.81 (0.42) 8.55 (0.3) | 10 (0.3) 8.46 (0.08) | 9.72 (0.44) 7.63 (0.23) | 10.39 (0.95) 6.83 (0.59) |
|  | June 20th, 2019 | (0-5)  (5-10) | **8.07 (0.16)** 7.06 (0.21) | **9.26 (0.32)** 6.91 (0.13) | 11.37 (0.52) 8.74 (0.13) | 11.12 (0.38) 9 (0.3) | **9.28 (0.41)** 7.09 (0.17) | **13.04 (1.54)** 6.27 (0.48) |
|  | August 8th, 2020 | (0-5)  (5-10) | 8.31 (0.17) 6.46 (0.11) | 7.99 (0.19) 6.75 (0.27) | 11.1 (0.25) **9.15 (0.22)** | 11.9 (0.41) **9.96 (0.22)** | 8.77 (0.31) 7.81 (0.59) | 9.4 (0.83) 6.01 (0.52) |
| Water extractable DOC and soil organic content (by loss in ignition) by site, treatment, depth, and date, reporting mean (standard error, n = 5). Bolding indicates a statistically significant difference between salt and control (one-way ANOVA, α = 0.05). | | | | | | | | |

**S2 Table (continued). Statistical summary.**

|  | |  | Site 1 | | Site 3 | | Site 5 | |
| --- | --- | --- | --- | --- | --- | --- | --- | --- |
|  | | Depth | Control | Salt | Control | Salt | Control | Salt |
| Phenolics (mg/L) | |  |  |  |  |  |  |  |
|  | May 10th, 2018 | (0-5)  (5-10) | 7.68 (0.51) 6.25 (0.85) | 6.34 (0.56) 6.85 (0.48) | 5.2 (0.62) 4.19 (0.37) | 6.17 (0.59) 4.57 (0.48) | 3.1 (0.46) 4.1 (0.78) | 4.27 (0.7) 5.77 (0.8) |
|  | July 10th, 2018 | (0-5)  (5-10) | **2.96 (0.13)** 3.45 (0.34) | **4.16 (0.39)** 3.59 (0.32) | 3.78 (0.35) 3.17 (0.3) | 4.54 (0.38) 2.96 (0.26) | 3.35 (0.14) **3.77 (0.27)** | 3.74 (0.24) **4.98 (0.41)** |
|  | June 20th, 2019 | (0-5)  (5-10) | 3.44 (0.3) 3.75 (0.24) | 3.16 (0.4) 3.16 (0.67) | **3.54 (0.22) 2.43 (0.14)** | **2.53 (0.14) 1.48 (0.2)** | 1.65 (0.13) 1.89 (0.14) | 1.98 (0.08) 2.52 (0.39) |
|  | August 8th, 2020 | (0-5)  (5-10) | 4.29 (0.41) **5.47 (0.36)** | 3.67 (0.17) **2.2 (0.42)** | **3.45 (0.32) 3.15 (0.49)** | **1.85 (0.32) 0.59 (0.09)** | **4.47 (0.68)** 3.03 (0.1) | **1.98 (0.32)** 2.87 (0.38) |
| Phenolics (mg/ mg DOC) | | |  |  |  |  |  |  |
|  | May 10th, 2018 | (0-5)  (5-10) | **0.39 (0.02)** 0.25 (0.07) | **0.25 (0.02)** 0.24 (0.03) | 0.22 (0.04) 0.22 (0.01) | 0.24 (0.01) 0.21 (0.01) | 0.35 (0.02) **0.39 (0.01)** | 0.3 (0.04) **0.3 (0.02)** |
|  | July 10th, 2018 | (0-5)  (5-10) | 0.18 (0) 0.17 (0) | 0.18 (0) 0.19 (0.01) | 0.2 (0.03) 0.21 (0.03) | 0.17 (0) 0.16 (0) | 0.18 (0.02) **0.15 (0.01)** | 0.22 (0.01) **0.22 (0.01)** |
|  | June 20th, 2019 | (0-5)  (5-10) | **0.18 (0) 0.16 (0)** | **0.16 (0) 0.15 (0)** | 0.15 (0.01) 0.13 (0) | 0.13 (0) 0.13 (0) | 0.13 (0) 0.12 (0.01) | 0.13 (0.01) 0.14 (0.01) |
|  | August 8th, 2020 | (0-5)  (5-10) | 0.15 (0) 0.15 (0.01) | 0.15 (0) 0.14 (0.01) | **0.12 (0) 0.12 (0.01)** | **0.1 (0.01) 0.08 (0.01)** | 0.15 (0.01) 0.15 (0.01) | 0.14 (0.01) 0.16 (0.01) |
| Water extractable soil phenolic content (total and per gram DOC) by site, treatment, depth, and date, reporting mean (standard error, n = 5). Bolding indicates a statistically significant difference between salt and control (one-way ANOVA, α = 0.05). | | | | | | | | |
